# New Insights into Milk and Dairy Products: Quality and Sustainability

**DOI:** 10.3390/foods13131969

**Published:** 2024-06-21

**Authors:** Paolo Formaggioni, Piero Franceschi

**Affiliations:** 1 Department of Veterinary Science, University of Parma, Via del Taglio 10, 43126 Parma, Italy; 2 Department of Food and Drug Science, University of Parma, Via delle Scienze 27/A, 43124 Parma, Italy

## 1. Introduction

The dairy industry is confronting a major challenge that could profoundly affect its future and its long-standing role as a cornerstone of human nutrition [1,2].

FAO, in its last report on the global trends, estimated an increase in the world’s population to almost 10 billion people by 2050 and thus, by 2050, agriculture must produce about 50% more food and feed than it did in 2013 [3]. Meeting the increased demand for food will not be the only challenge; indeed, beyond producing food and feed, the agriculture and food industries will have to produce it in a sustainable way [4].

Therefore, the primary mission will lie in providing adequate solutions to meet the demand for nutritionally balanced and environmentally, economically and socially sustainable products [5]. To this aim, generally, ensuring the safety of dairy products is the most important requirement. However, the quality of milk and its derived products is crucial. For instance, the capacity of milk to coagulate with rennet is essential for cheese production, as it impacts both the yield and the quality of the cheese [6]. In addition to this, rheological and microbiological properties are significant for obtaining dairy products that serve various purposes [6]. Therefore, gaining new insights into how genetic, physiological, pathological, environmental, and technological factors influence the quality of milk and dairy products will contribute to the progress of the sector.

Furthermore, milk and dairy products are vital sources of nutrients for humans, providing proteins, fats, calcium, and vitamins. The dairy industry also produces several by-products, such as whey and buttermilk, which are valuable, due to their high nutritional content and can be repurposed for other uses [7]. This repurposing also helps in reducing the environmental pollution caused by the industry [7].

In addition, milk from certain species has not been extensively studied or characterised; this untapped potential could be used to create new dairy products.

## 2. An Overview on the Published Articles

This Special Issue, titled “New Insights into Milk and Dairy Products: Quality and Sustainability”, has collected 19 articles (14 original research studies and 5 reviews) that make significant contributions to the field. These articles can be categorised into four main groups: the first category includes studies that address the microbiological aspects of milk, cheeses, and other dairy products; the second category has a focus on product innovation, featuring seven studies that discuss technological advancements and compositional aspects related to process improvements, new milk coagulants, and novel dairy foods; the third category regards papers in which genetics are applied to milk quality, by means of identification of functional genes or markers; the fourth category includes three reviews and an original study addressing various and base aspects of dairy science.

*Microbiological aspects of milk, cheeses, and dairy products*. Alsulami et al. [contribution 1] focused their study on the sustainability of camel milk, a nutritious dairy product widely consumed in the Middle East, particularly in Saudi Arabia. The study addressed the major concern of aflatoxin contamination in camel milk. Samples from the Arabian Peninsula and North Africa were analysed for aflatoxins B1 and M1 by means of ELISA and fluorometer methods. The results showed significant aflatoxin M1 contamination and cross-contamination with aflatoxin B1. This study also demonstrated the effectiveness of two probiotic bacteria strains, *Lactobacillus plantarum* NRC21 and *Lactobacillus acidophilus* NRC06 (both categorised as probiotic strains), in inhibiting toxigenic fungi and reducing aflatoxin levels in both liquid media and spiked samples of camel milk. The findings suggest that fermenting camel milk with these probiotic strains could be an effective method for reducing aflatoxin contamination.

Idland et al. [contribution 2] also address hygienic–sanitary issues and their impact on human health. In particular, they investigated the presence and proliferation of Shiga toxin-producing *Escherichia coli* (STEC) in raw milk, considering its association with human illnesses like diarrhoea, haemorrhagic colitis, and haemolytic uremic syndrome. The pathogenicity of STEC is linked to its production of Shiga toxin (Stx) and its capability to attach to the intestinal epithelium through the adhesion protein intimin, which is encoded by the eae gene (intimin-encoding gene). The authors studied four eae-positive STEC isolates from Norwegian dairy herds, analysing their ability to grow in unpasteurised milk (UPM) under various temperature conditions. Genome analysis indicated that three of the isolates were clonal, suggesting transmission between farms. All isolates produced Shiga toxin, with one capable of growing at 8 °C, posing a significant health risk. This study suggests the need for better consumer awareness and proper refrigeration practices; moreover, even properly stored unpasteurised milk poses an increased risk of illness, especially for vulnerable populations like young children, the elderly, and immunocompromised individuals.

Papadimitriou et al. [contribution 3] investigated the microbiome of Staka (a Cretan sour cream) utilising various methods, including culture-based techniques, amplicon sequencing, and shotgun metagenomics. Staka is a traditional Greek cultured cream made from spontaneously fermented sheep’s milk or a mixture of sheep’s and goat’s milk. The study revealed that the samples were predominantly composed of *Lactococcus* and *Leuconostoc* species, along with other bacteria such as *Streptococcus* and *Enterococcus*, and Gram-negative genera like *Enterobacter*, *Pseudomonas*, *Buttiauxella*, *Escherichia-Shigella*, and *Hafnia*. They also found common genera of yeasts and moulds. Through shotgun metagenomics, specific species were identified. This study also isolated novel strains from Staka with antibacterial potential. Furthermore, several LABs, *Hafnia paralvei* and *Pseudomonas* spp., have antimicrobial activity against pathogenic bacteria, indicating their potential use in food safety and biomedical applications.

Tsouggou et al. [contribution 4] examined the microbiome of industrial PDO Sfela cheese and its artisanal variants utilising 16S rDNA amplicon sequencing and shotgun metagenomics, to better understand its complex microbial ecosystem, which is crucial for sustainably enhancing production and safety measures. Sfela, a white, salted PDO Greek cheese from the Peloponnese region, is made from sheep’s milk, goat’s milk, or a mixture of both. The study examined two PDO industrial Sfela samples and two non-PDO variants (Xerosfeli and Sfela touloumotiri) using MALDI-TOF MS, 16S rDNA amplicon sequencing, and shotgun metagenomics. Analysis with culture media revealed the presence of *Lactiplantibacillus plantarum*, *Enterococcus faecium*, *Pediococcus pentosaceus*, *Levilactobacillus brevis*, and *Streptococcus thermophilus* as the most common bacterial species. Shotgun analysis revealed *Streptococcus thermophilus* dominance in industrial Sfela 1 and high levels of *Lactococcus lactis* in industrial Sfela 2. The samples of artisanal Xerosfeli and Sfela touloumotiri were mainly composed of *Streptococcus thermophilus* and *Tetragenococcus halophilus*, respectively. Additionally, *Debaryomyces hansenii* was the only yeast present in quantities exceeding 1%, and was detected only in Sfela touloumotiri samples.

Sun et al. [contribution 5] investigated the relationship between the microbiota present in raw milk and that found in the resulting cheese, specifically a Swedish long-ripened cheese, with the aim of understanding why cheeses generally take longer to mature now than in the past. Three commercial farm clusters were created to introduce variability in the microbiota of dairy silo milk utilised for the production of cheese. This latter process took place in three different periods throughout the year, with milk from each farm cluster being collected separately, pasteurised, and processed into cheese. Samples were collected at different stages, including herd bulk milk and dairy silo milk, starting cultures, early processing samples and samples of cheese at various maturation stages (7–20 months) and analysed by means of 16S rRNA amplicon sequencing. The microbiota in herd bulk milk varied significantly among periods and among clusters, while the microbiota in dairy silo milk showed differences only across periods. The microbiota in cheese differed by periods and groups, with *Leuconostoc* and *Lactococcus* emerging as the predominant genera in both early processing and samples of cheese. Surprisingly, the abundance of *Lactobacillus* was very low during cheese ripening, and even at later stages starter lactic acid bacteria were dominant.

The review by Silva et al. [contribution 6] discusses the microbiological quality of cheese made from raw milk. This review focuses specifically on alternative systems for milk sanitisation other than pasteurisation. Plant extracts and lactic acid bacteria seem to offer promising methods for minimising microbial contamination in artisanal raw milk cheese. This is attributed to their natural components, like phenolic compounds in plant extracts, and the capacity of lactic acid bacteria to produce antimicrobial substances such as organic acids and bacteriocins. Furthermore, thermisation is considered an alternative strategy to pasteurisation. It aims to preserve the sensory qualities of artisanal cheeses while also effectively inactivating microorganisms by disrupting their cellular structure and functions. This review explores the antimicrobial mechanisms, benefits, drawbacks, and practical applications of all three strategies.

*Process technological improvements, new milk coagulants, and novel foods*. The papers in this section can in turn be grouped into three subsections, each constituted by two studies: high pressure as a sanitising process for milk or cream; new vegetable coagulants for milk; the addition of vegetable to milk to obtain novel foods. The six papers are presented in this order.

Lim et al. [contribution 7] investigated the impact of high-pressure processing (HPP) for 10 min at 600 MPa on the quality, safety, and shelf life of raw milk stored at 6 °C for 60 days. This method provides an alternative to thermal processing and preserves the nutritional integrity of raw milk. HPP-treated milk satisfied all microbiological safety standards and demonstrated a shelf life exceeding 60 days, even under hot and humid conditions. Additionally, it effectively preserved nearly all vitamins and minerals, including phosphorus (99.4%), calcium (99.3%), and magnesium (99.1%). However, over the 60-day storage period, there was partial degradation of vitamins A (25%), B3 (91%), B5 (35%), B6 (80%), and C (85%), as well as minerals like zinc (18%) and potassium (5%), compared to fresh milk. This study highlights the significant benefits of adopting advanced HPP processing technology in the dairy industry for maintaining the physico-chemical and nutritional properties of milk and extending its shelf life beyond 60 days.

The study by Machado et al. [contribution 8] also evaluated the effects of high-pressure processing (HPP) at 450 and 600 MPa for 5 and 15 min at 7 °C, compared to thermal pasteurisation at 75 °C for 15 s, on the microbiological and physico-chemical quality of dairy cream. The assessments were made both immediately after processing and during refrigerated storage at 4 °C. HPP-treated samples were still microbiologically safe even after 51 days of storage, unlike samples treated with thermal pasteurisation. HPP effectively reduced populations of *Escherichia coli* and *Listeria innocua* by more than 6 log units to undetectable levels (1.00 log CFU/mL). The pH, colour, and fatty acid profiles of the cream remained stable under various processing conditions and throughout storage. Furthermore, there was a tendency for volatile compounds (VOCs) to increase in all treated samples during storage, especially acids and aliphatic hydrocarbons. These findings suggest that HPP can significantly extend the shelf life of highly perishable dairy cream by at least 15 days compared to thermal pasteurisation.

Bande-De-Léon et al. [contribution 9] studied the effect of two novel plant milk coagulants (freeze-dried extracts from *Cynara humilis* L. (CH) and *Onopordum platylepis* Murb. (OP)) and compared their coagulation and proteolytic activities with those of *Cynara cardunculus* L. (CC). They examined the impact of extract concentration (5–40 mg/mL), pH (5–8), temperature (20–85 °C), and CaCl_2_ concentration (5–70 mM) on the milk coagulation activity (MCA) of CC, CH, and OP extracts. At the same concentration, CC exhibited significantly higher MCA values. Among these various extracts, the clotting activity of OP increased with increasing temperature, reaching a maximum value at 70 °C. Adding CaCl_2_ improved the coagulation ability of the extracts, particularly for OP and CH. Additionally, the proteolytic activity and the hydrolysis rate increased over time and with higher enzyme concentrations, with CC showing the highest caseinolytic activity among all the extracts.

Nicosia et al. [contribution 10] studied the use of kiwi fruit aqueous extract as a cheese coagulant. In particular, they used SDS-PAGE to detect actinidin, the kiwi enzyme responsible for the hydrolysis of casein, in various parts (pulp, peel, and whole fruit) of both ripe and unripe kiwi fruits. Actinidin was present in both the peel and the pulp. While the peel extract could partially hydrolyse skimmed milk, it could not degrade casein. On the contrary, the pulp extract demonstrated hydrolytic activity against both the casein and the skimmed milk. Ripe kiwi extracts showed higher hydrolytic activity than unripe ones. Using a 3% (*v*/*v*) extract from ripe kiwi pulp resulted in a curd yield of 20.27%, similar to that achieved with chymosin. In summary, the extraction method for the aqueous kiwi extract is fast, economical, chemical-free, and environmentally friendly, making it an effective vegetable coagulant for cheese production.

Semeniuc et al. [contribution 11] developed a Gouda-type cheese from cow milk, flavoured with pollen of lavender (0.5 g/L of mature milk), and aged for 30 days at 14 °C, with 85% of relative humidity. They evaluated the physico-chemical, microbiological, and textural properties of the lavender cheese and control cheese at 10-day intervals. Lavender pollen significantly influenced the sensory and microbiological properties, as well as the volatile compounds of the cheese, but had a modest impact on its physico-chemical and textural properties. During the ripening process, the moisture, carbohydrate contents, pH, viscosity, elasticity, and chewiness of both cheeses decreased, while increases were observed for protein content, titratable acidity, ash, sodium chloride content, hardness, streptococci, lactobacilli, and volatile compounds. Despite lavender’s known antibacterial effects against *Escherichia coli* and *Clostridium butyricum*, it did not inhibit the growth of microorganisms of the starter culture; instead, it promoted the growth of lactic acid bacteria. Sensory evaluations revealed that the overall score for the lavender cheese was slightly lower but comparable to the control cheese, with which consumers were more familiar.

In their review, Lima Nascimento et al. [contribution 12] examine the integration of milk proteins with plant proteins, as a strategy to address the functional and sensory limitations of plant proteins. This combination results in various colloidal systems, such as suspensions, emulsions, gels, and foams, commonly found in food products. For example, adding plant protein can solve technical problems, like preventing gel formation in high-protein milk drinks during packaging. Dairy proteins can also improve the solubility of certain plant proteins in dispersions of mixed protein. By understanding the technical and functional properties of these proteins and their responses to environmental conditions, such as temperature and pH, new products like yogurt, analogues of cheese, and beverages with desired textures and sensory properties can be developed. To further optimise these systems and their applications in the food industry, innovative techniques to modify the technical and functional characteristics of both plant and dairy proteins, like pulsed electric fields, precision fermentation, and high hydrostatic pressure, are being evaluated.

*Genetics applied to milk quality: identification of functional genes or markers.* The synthesis of milk protein is regulated by a complex network involving numerous signalling pathways. Chen et al.’s [contribution 13] study aimed to elucidate these pathways in goat mammary epithelial cells (GMECs) utilising microproteomic techniques, and to identify the key genes involved. Their analysis identified over 2253 proteins and annotated 323 pathways. This study highlighted the significant role of the IRS1 (insulin receptor substrate 1) gene in influencing casein content in goat milk and the pathways that are involved in milk protein synthesis in GMECs. Altering IRS1 expression increased the amounts of β-casein and κ-casein but decreased α-casein levels. By identifying proteins that, in response to IRS1 silencing, were differentially expressed, the researchers gained new insights into the network and signalling pathways associated with goat milk protein synthesis. These findings could lead to new strategies for modifying the content of casein in the dairy goat sector and for developing milk products.

Yang et al. [contribution 14] studied the polymorphisms in the CCSER1 gene of Gannan yaks, identifying three SNP loci and analysing their relationship with milk quality. The study found that all three SNPs showed moderate polymorphism. The analysis of correlations revealed that the mutant genotype at the CCSER1 g.183,843A>G locus significantly increased milk fat content (*p* < 0.05). Additionally, mutant genotypes at the CCSER1 g.222,717C>G and g.388,723G>T loci significantly increased both casein and protein contents in milk from Gannan yak (*p* < 0.05). Thus, mutations at these loci (g.183,843A>G, g.222,717C>G, and g.388,723G>T) notably improved the quality of milk from Gannan yak. The identification of these SNPs can allow the development of further research and application in selecting Gannan yaks, helping in the improvement and optimisation of their milk quality.

The study by Ma et al. [contribution 15] also investigated the polymorphism of Gannan yak genes. In particular, they specifically focused on the PRKD1 and KCNQ3 genes. This was a pioneering study examining the connection between these gene polymorphisms and dairy traits; in particular, it evaluated the relationship between the single nucleotide polymorphisms (SNPs) of these two genes and the milk quality of Gannan yaks, aiming to identify potential molecular markers for breeding. Using a technology Illumina yak cGPS 7K liquid-phase microarray, they detected, in 172 lactating Gannan yaks, three new SNPs: two in the PRKD1 gene (g.283,619T>C, g.283,659C>A) and one in the KCNQ3 gene (g.133,741T>C). Association analysis revealed significant correlations between these gene polymorphisms and milk quality traits. Specifically, mutations at these loci were found to significantly improve the levels of fat, lactose, protein, casein, non-fat milk solids (SNF), and acidity in Gannan yak milk. Therefore, genotyping the PRKD1 and KCNQ3 genes can effectively improve milk quality in Gannan yaks.

*Reviews and experimental studies on general aspects of dairy.* Franceschi et al. [contribution 16] studied the distribution between the colloidal and soluble phases of calcium, phosphorus and magnesium, and their level within yak milk casein micelles. They compared nine samples of yak milk from Qinghai, China, with nine bulk cow milk samples. The authors found similar levels of colloidal calcium, higher levels of magnesium, and lower levels of colloidal phosphorus per casein unit in yak milk. Yak milk was characterised by high casein and mineral content, and was particularly rich in colloidal forms of Ca, P, and Mg, which could enhance the bioavailability of calcium and phosphorus during digestion. Moreover, yak milk had lower prosthetic phosphorus per casein unit, suggesting less phosphorylated amino acids, which may affect the micelle structure, as well as the processability and the digestion of yak milk casein in comparison to cow milk casein.

The aim of the systematic review of Castellini et al. [contribution 17] was to examine and categorise the conceptual attributes of milk quality, as perceived by citizen consumers, farmers, and processing experts. Using PRISMA guidelines, they screened 409 papers and assessed 20 full-text papers. This review identified 12 main attributes defining milk quality, the most prominent of which were nutritional quality/healthiness, hygienic quality, and knowledge and attitudes of workers. Farmers and processing experts had similar technically focused perceptions of milk quality, emphasising expertise and knowledge. On the contrary, citizen consumers gave a representation of milk quality in more simplistic and subjective terms. This study underlined the need to create a common platform for communication and exchange of knowledge, to align the different perceptions and expectations of milk quality.

The review by Martuzzi et al. [contribution 18] summarises recent research on microorganisms in fermented products from mare milk and their potential functional properties. Mare milk is consumed by approximately 30 million people worldwide and is primarily used in Asia and Eastern Europe to produce fermented and alcoholic beverages like koumiss, airag, or chigee, made using bacteria and lactose-fermenting yeast cultures. The review details the main bacterial and yeast species found in these products, highlighting a complex population that includes lactic acid bacteria, yeasts, acetic acid bacteria, and white moulds. The focus of this review is on the potential health benefits and functional properties of these mare milk products, making them highly nutraceutical foods, with the aim of optimising their use in industrial production, particularly for koumiss.

The review by Linehan et al. [contribution 19] compared milk production, nutritional, and compositional properties between conventional and organic dairy systems, highlighting the health benefits of organic milk and the global landscape of organic dairy production. During the past two decades, organic dairy has increased in importance, due to concerns over the use of antibiotics, fertilisers, and pesticides, as well as animal health, and increasing environmental and self-health awareness. Most reports suggest that milk generally has beneficial effects on human health, with few, if any, negative effects. Organic milk offers some further benefits, due to a lower omega-6 to omega-3 ratio, attributed to pasture-based practices of feeding. However, organic milk production can be difficult in some regions, due to high costs and geographical conditions. The review also highlighted future perspectives and identified knowledge gaps in organic dairy management.

## 3. Further Remarks and Conclusions

An interesting aspect of this Special Issue is that contributions related to milks other than cow’s milk have also been included: camel milk [contribution 1], mare milk [contribution 18], yak milk [contributions 14–16], goat milk [contribution 13], goat milk, sheep milk, or a mixture of goat and sheep milk [contributions 3,4].

Overall, this Special Issue “New Insights into Milk and Dairy Products: Quality and Sustainability” presents readers with a wealth of innovative information that can serve as a valuable resource for sustainability, for generating new research ideas and developing novel dairy products.

To conclude, we want to express our gratitude to the research teams mentioned earlier for their valuable contributions to this collection of articles present in this Special Issue. Their studies demonstrate the diverse and interdisciplinary nature of cheese and dairy research.

## Data Availability

The data presented in this study are available on request from the corresponding author.
